# A Case of Adult-Onset Still's Disease With Positive Antinuclear Antibodies and Positive Antineutrophil Cytoplasmic Antibodies

**DOI:** 10.7759/cureus.61399

**Published:** 2024-05-30

**Authors:** Valentin Vasilev, Maria Savvina, Agrafena Argunova, Aitalina Danilova

**Affiliations:** 1 Department of General Medicine, Medical Institute of the Federal State Autonomous Educational Institution of Higher Education, M.K. Ammosov North-Eastern Federal University, Yakutsk, RUS; 2 Department of Internal Medicine, State Budgetary Institution of the Republic of Sakha (Yakutia) Yakutsk Republican Clinical Hospital, Yakutsk, RUS; 3 Department of Hospital Therapy, Occupational Diseases, and Clinical Pharmacology, Medical Institute of the Federal State Autonomous Educational Institution of Higher Education, M.K. Ammosov North-Eastern Federal University, Yakutsk, RUS; 4 Department of Administration, Clinic No. 1, State Autonomous Institution of the Republic of Sakha (Yakutia), Yakutsk, RUS

**Keywords:** ana positive aosd, anca positive aosd, papular rash, follow-up monitoring, yakut nationality, russia, paracetamol, cytomegalovirus, salmon-colored rash, case report

## Abstract

Adult-onset Still's disease (AOSD) is a rare autoinflammatory disease characterized by nonspecific symptoms such as fever, maculopapular rash, and arthralgias. The exact etiology and pathogenesis remain unclear despite advancements in medical science. Diagnosis is typically established using the Yamaguchi criteria, which include a negative antinuclear antibody (ANA) test as one of the minor criteria. However, some patients with AOSD exhibit positive ANA and even positive antineutrophil cytoplasmic antibodies (ANCA), complicating the diagnostic process.

We present the case of a 19-year-old Asian woman of Yakut ethnicity who initially presented with symptoms resembling an upper respiratory tract infection. Laboratory tests revealed the presence of both ANA and ANCA. The diagnosis of AOSD was confirmed based on clinical presentation and the Yamaguchi criteria. Subsequent pulse therapy with prednisolone resulted in significant clinical improvement and a one-year remission. A review of the literature revealed that simultaneous ANCA and ANA positivity in AOSD has not been previously reported. Follow-up over 12 months showed no evidence of other autoimmune or autoinflammatory diseases, suggesting that the positive ANA and ANCA results may be either false positives or atypical laboratory manifestations in AOSD, which should be considered in the diagnosis.

## Introduction

Adult-onset Still's disease (AOSD) is a rare autoinflammatory disease with a prevalence ranging from 0.16 to 3.9 per 100,000 people [1]. The etiology of AOSD remains unknown, although it is hypothesized that various infectious agents may act as triggers. The pathogenesis is also not precisely understood, but it is thought to involve excessive secretion of IL-1β and IL-18 in individuals with an unknown predisposition [2]. Diagnosis of AOSD is challenging due to the lack of specific markers and typically occurs 1-4.6 months after the onset of initial symptoms [1].

The clinical presentation of AOSD is limited and primarily includes nonspecific symptoms such as fever, a characteristic maculopapular rash, and arthralgias. The Yamaguchi criteria are most commonly used for diagnosis due to their high sensitivity and ease of application compared to other diagnostic criteria [3]. One of the minor Yamaguchi criteria is a negative antinuclear antibody (ANA) level [4]; however, positive ANA can occur in 2.9%-25.8% of patients [1]. The role of ANA positivity in the pathogenesis of AOSD remains unclear; there are no studies on the clinical course of the disease in such patients, and the limited number of cases described does not provide a comprehensive understanding. Regarding ANCA-positive patients, there are no data in the literature describing cases of AOSD with this serological marker.

This report presents a case of AOSD in a patient positive for both ANA and ANCA, with a 12-month follow-up to exclude other autoimmune diseases.

## Case presentation

**Figure 1:**
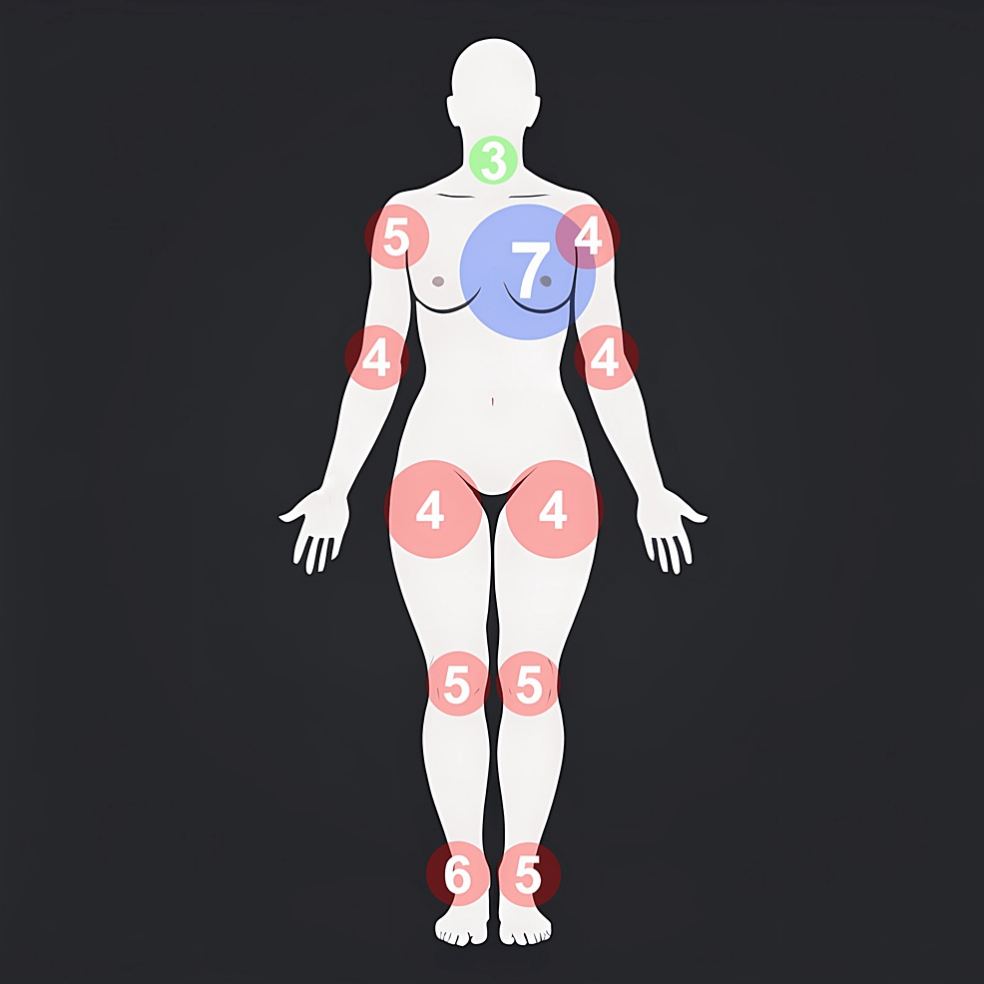
Localization and level of pain at the time of admission to the city hospital. The color red indicates pain in the joints, blue indicates pain in the heart area, and green indicates pain in the throat. The pain level is indicated on a visual analog scale (0 points = no pain, 10 = worst possible pain).

The patient was a 19-year-old Asian woman of Yakut nationality. She was admitted to the Department of Internal Medicine with complaints of dyspnea, compressive chest pain, joint and muscle pain, sore throat, palpitations, nocturnal fevers reaching up to 40 °C, weakness, diarrhea, decreased appetite, frequent urges to urinate, and a weight loss of 5 kg over the past month (Figure 1). On examination, there was a slight hyperemia of the posterior pharyngeal wall.

**Figure 2:**
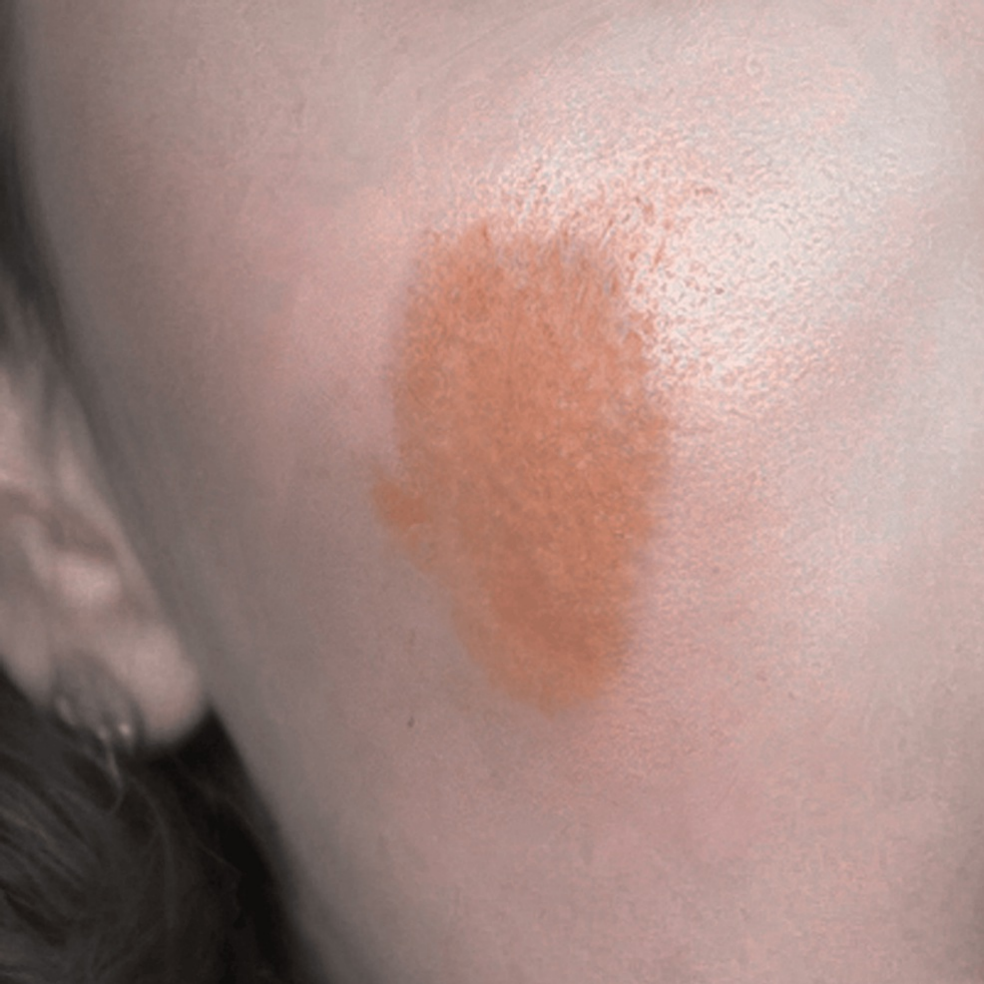
The only surviving photograph of a 1 cm × 2 cm macular rash on the right cheek, taken from the patient's personal album. All other photos were lost.

One month before hospitalization, the patient experienced severe hypothermia following a bath. The next day, she developed a sore throat and a fever of 37 °C, which increased to 40 °C at night. One day later, she developed pain in the muscles and joints of her limbs, with symptoms worsening during episodes of fever. The patient also noted a painless, maculopapular focal salmon-colored rash measuring 0.5 cm × 0.5 cm on her right thigh and a 1 cm × 2 cm papular rash on her right cheek, both of which resolved without a trace after six hours (Figure 2). The rash on the cheek was notable for rapidly growing to a height of approximately 0.5 cm above the skin surface. The patient initially believed this rash was an allergic reaction to ketorolac, which she was taking at a dose of 30 mg daily to reduce fever, so she did not report it.

The patient received treatment from her physician for a suspected upper respiratory tract infection, consisting of levofloxacin at a dosage of 1,000 mg per day and paracetamol up to 4,000 mg per day, resulting in symptom improvement and a drop in temperature to 37 °C. However, upon discontinuation of paracetamol, her temperature rebounded to 40 °C, accompanied by a recurrence of other symptoms. She did not revisit the doctor. Independently, she resumed paracetamol, achieving a reduction in temperature to 36 °C, yet other symptoms persisted. She was taking a dose of 4,000 mg daily.

Gradually, her condition worsened, and she decided to see a doctor in the hospital. Upon hospitalization in the Department of Internal Medicine, laboratory tests were performed (Table 1). A polymerase chain reaction (PCR) test for COVID-19 was negative. An electrocardiogram (ECG) revealed tachycardia with a heart rate of 122 beats per minute. Ultrasound showed a small effusion in the pericardial cavity, a small effusion in the right pleural cavity, and hepatosplenomegaly. On admission, there was slight leukocyturia and the presence of mucus in the urine. Acute pyelonephritis with systemic inflammatory response syndrome and iron deficiency anemia were suspected. The patient was treated with imipenem, cilastatin, and diclofenac, and received two transfusions of erythrocyte suspensions. Despite therapy, the fever persisted. A CT scan of the abdomen with contrast excluded acute pyelonephritis. Further diagnostics, including thyroid ultrasound, brain CT scan, esophagogastroduodenoscopy, irrigoscopy, and rectosigmoidoscopy, did not reveal any pathology.

**Table 1:** Basic laboratory data. ANC, absolute neutrophil count; ESR, erythrocyte sedimentation rate; GGTP, gamma-glutamyl transpeptidase; LDH, lactate dehydrogenase; ALP, alkaline phosphatase; CRP, C-reactive protein; IL-6, interleukin-6

Parameter	Hospitalization	After initiation of prednisolone therapy	Six months after discharge	12 months after discharge	Reference range
White blood cells	14.5	9.8	15.6	5.6	4-10 x 10^9^/L
Hemoglobin	78	115	135	129	110-160 g/L
ANC	11.6	7	13.1	-	2-7 x 10^9^/L
Platelets	809	359	357	371	150-400 х 10^9^/L
ESR	26	10	23	20	5-18 mm/hr
Albumin	29.7	35	-	46	38-54 g/L
Creatinine	31	29	30	45	44-80 µmol/L
GGTP	224.4	-	-	13	0-32 U/L
LDH	288	215	-	-	135-214 U/L
ALP	445.9	194	59	120	35-104 U/L
CRP	141.3	7.9	-	6.1	0-5 mg/L
Serum iron	6.4	19.5	19.3	14.5	6.6-26 µmol/L
Ferritin	6,375	1,309	240	-	10-120 mcg/L
Fibrinogen	10.5	4.7	1	5.1	2.4-4.3 g/L
D-dimer	1006	2359	-	0	0-250 ng/mL
Antistreptolysin O	-	-	300	299	0-200 IU/mL
IL-6	35.5	-	-	-	1.3-6.8 pg/mL
Leukocytes in urine	20	1	2	2	0-5 per high-power field
Mucus in urine	Positive	Negative	Negative	Negative	Negative

After ruling out infectious diseases with extensive laboratory tests, we began to consider the possibility of AOSD (Table 2). Other rheumatologic diseases did not align with the laboratory and clinical findings, but the presence of positive ANA and ANCA results, combined with the absence of rash during the clinical examination, caused some initial confusion. We conducted a detailed interview with the patient, who then recalled experiencing a rash at the onset of the disease and provided photographs. The patient had assumed the rash was an allergic reaction, so she had not reported it to the medical staff. The characteristic rash, along with a clear clinical picture, confirmed the diagnosis of AOSD.

We initiated pulse therapy with prednisolone, starting with 120 mg on the first day, followed by 90 mg on the second day, and 60 mg on the third day, before transitioning to methylprednisolone 20 mg daily. The patient showed immediate improvement, with normalization of temperature and favorable changes in laboratory values (Table 1). A subsequent ultrasound showed slight hepatomegaly without fluid accumulation in the pericardium or pleural cavity. The patient was discharged with instructions for outpatient treatment, including methylprednisolone 20 mg daily and methotrexate 10 mg weekly.

**Table 2:** Additional laboratory data. Polymerase chain reaction (PCR) of oropharyngeal swab: PCR of oropharyngeal swab for adenovirus, bocavirus, influenza A virus, influenza B virus, parainfluenza virus, SARS-CoV-2, coronavirus (229E, OC43, NL63, and HKU1), metapneumovirus, respiratory syncytial virus, rhinovirus, and Streptococcus pyogenes. HCV, hepatitis C virus; HBsAg, hepatitis B surface antigen; ELISA, enzyme-linked immunosorbent assay; ANCA, antineutrophil cytoplasmic antibodies

Parameter	Result
Blood group ABO/Rh blood group/Kell antigen	А/Rh+, DCCCee/Negative
HIV-1, HIV-2 rapid screen	Negative
PCR blood test for herpes simplex virus, Epstein-Barr virus, cytomegalovirus	Negative
Rapid Plasma Reagin	Negative
Anti-HCV	Negative
HBsAg	Negative
Herpes simplex virus 1, 2 IgG antibodies	Positive
Herpes simplex virus 1, 2 IgM antibodies	Negative
Cytomegalovirus IgG antibodies	Positive
Cytomegalovirus IgM antibodies	Positive
Coombs test (direct and indirect)	Negative
Cyclic citrullinated peptide antibodies	Negative
Anti-double-stranded DNA antibodies	Negative
Antinuclear antibodies (ELISA)	Positive
Antineutrophil cytoplasmic antibodies (p-ANCA, c-ANCA)	Positive
Anti-centromere antibodies	Negative
PCR of oropharyngeal swab	Negative
Sputum culture	Negative
Blood culture	Negative
Fecal occult blood	Negative
Microscopic examination of feces	Negative
Microscopic examination of urine for acid-fast bacteria	Negative
Mantoux test	Negative

Follow-up examinations at six and 12 months, including laboratory tests, showed that at the first visit, the patient reported a slight sore throat and had discontinued medication due to a seven-week pregnancy (Table 1). Due to the risk of complications from recent methotrexate intake, an abortion was performed. The patient chose to discontinue medication, believing she was in remission. At the second follow-up, she reported no complaints but had a high ASLO level of up to 299 IU/mL, despite denying any infections. We advised to undergo repeat laboratory evaluation for the presence of ANCA and ANA, but the patient refused. Examinations that consistently fell within reference values from the initial hospitalization to the last follow-up are listed separately (Table 3).

**Table 3:** Laboratory data consistently remained within reference values from disease onset to the last follow-up examination.

Parameter	Reference range
Red blood cell	3.5-5.5 x 10^12^/L
Absolute lymphocyte count	0.8-4.0 x 10^9^/L
Absolute monocyte count	0.04-0.7 x 10^9^/L
Absolute basophil count	0-0.1 x 10^9^/L
Absolute eosinophil count	0-0.5 x 10^9^/L
Total protein	65-85 g/L
Glucose	3.3-5.8 µmol/L
Uric acid	0-340 µmol/L
Urea nitrogen	0-8.3 mmol/L
Total cholesterol	0-5.2 mmol/L
High-density lipoprotein	0.7-2.28 mmol/L
Low-density lipoprotein	0-2.5 mmol/L
Triglycerides	0-2.25 g/L
Conjugated bilirubin	0-3.4 µmol/L
Unconjugated bilirubin	0-16.4 µmol/L
α-Amylase	28-100 U/L
Alanine aminotransferase	0-31 U/L
Aspartate aminotransferase	0-32 U/L
Creatine kinase	0-145 U/L
Creatine kinase MB	0-25 U/L
Myoglobin	Negative
Procalcitonin	0-0.49 ng/mL
Troponin I	Negative
Na	136-145 µmol/L
K	3.5-5.1 mmol/L
Ca	2.3-2.8 µmol/L
Mg	0.66-1.7 mmol/L
Complement C3	0.9-1.8 g/L
Complement C4	0.1-1.4 g/L
IgA	0.61-3.48 g/L
IgG	5.49-15.84 g/L
IgE	0-100 IU/mL
IgM	0.23-2.59 g/L
Rheumatoid factor	0-14 IU/mL
Partial thromboplastin time	25-35 seconds
International normalized ratio	0.9-1.1
Alpha-fetoprotein	0-5.8 IU/mL
Carbohydrate antigen 19-9	0-34 U/mL
Mucin-16	0-35 U/mL
Carcinoembryonic antigen	0-5.5 ng/mL
Free triiodothyronine	3.1-6.8 pmol/L
Free thyroxine	10.3-24.5 pmol/L
Thyroid peroxidase antibodies	0-34 IU/mL
Thyroid-stimulating hormone	0.51-4.3 mcIU/mL
Protein in urine	0-0.15 g/L
Glucose in urine	0-0.8 mmol/L
Ketones in urine	0-0.06 mmol/L
Blood in urine	Negative
Bilirubin in urine	Negative
Urobilinogen in urine	0-3.4 µmol/L
Nitrites in urine	Negative

## Discussion

The time to correct diagnosis from the onset of the first symptoms in our case was 52 days. AOSD was diagnosed based on the Yamaguchi criteria [4], with lymphadenopathy and negative ANA and rheumatoid factor results excluded from all criteria (Table 4).

**Table 4:** Yamaguchi diagnostic criteria. Five or more criteria are required, of which at least two must be major criteria. Typical rash: An evanescent, salmon-colored, macular, or maculopapular cutaneous eruption that is usually nonpruritic and tends to occur with fever, disappearing during the febrile episode.

Major criteria	Minor criteria
Fever of at least 39 °C lasting at least one week	Sore throat
Arthralgias or arthritis lasting two weeks	Lymphadenopathy
Typical rash	Hepatomegaly or splenomegaly
Leukocytosis > 10,000, with at least 80% granulocytes	Abnormal liver function tests
	Negative tests for antinuclear antibody and rheumatoid factor

In this clinical case, the positive ANCA and ANA results (the titer of which, unfortunately, could not be established in our laboratory) are particularly noteworthy. Positive ANA is a marker in autoimmune diseases found in 20% to 30% of the healthy population [5]. Positive ANCAs are generally markers of diseases associated with small-caliber necrotizing vasculitis [6]. To rule out ANCA-associated disease, we followed the patient for a year but found no evidence of disease onset according to the laboratory and clinical data presented. ANA-positive cases of AOSD have been published previously [7-10], but their limited number and nonstandardized laboratory tests make it difficult to draw definitive conclusions. Positive ANCAs in AOSD may be an epiphenomenon, as suggested by one study [11]. However, due to the scarcity of research on this topic, definitive statements cannot be made. A PubMed database search from 1975 to 2024 using the keywords "adult-onset Still's disease," "AOSD," "positive antineutrophil cytoplasmic antibodies," and "positive ANCA" revealed no similar cases like ours.

The rash elements in AOSD are typically transient, salmon-colored, maculopapular, and predominantly appear on the extremities. However, multiple cases of atypical rashes have been reported in the literature [12]. In our case, the rash on the right thigh was a classic salmon-colored, multiple papular rash, but the rash on the right cheek was a significantly elevated, irregular, dark orange-colored bump at its peak. It is worth noting that we only saw this rash in the photographs provided by the patient. Since these photographs were lost, we cannot be sure of the color reproduction accuracy or if the photos were distorted. Nevertheless, we report this peculiarity of the AOSD course, as the patient was confident about the unusual morphology of the rash.

Initially considering the disease to be of infectious origin, we performed extensive analyses to identify a possible causative agent. We hypothesize a potential trigger for the onset of AOSD based on the elevated titer of antistreptolysin O, which may indicate a streptococcal infection. However, our result may be a false positive because we did not perform anti-DNase testing and cannot confirm that it was not a cross-reaction with other streptococcal species [13]. Interestingly, the patient maintained high antistreptolysin O titers up to 300 IU/mL for a year after recovery and without a sore throat clinic. The patient reported no prior complaints of a sore throat or episodes of pharyngitis or tonsillitis in her medical records. Unfortunately, we could not perform repeat microflora studies due to lack of resources, but prolonged elevated titers for more than a year have been noted in other studies [14]. Whether this is a peculiarity of AOSD or a result of bacterial carriage. Another potential trigger could be cytomegalovirus, as positive titers of IgG and IgM were detected. One study suggests a link between cytomegalovirus and AOSD [15], proposing that recurrent cytomegalovirus infection may enhance innate immunity, leading to AOSD. However, no direct causal relationship has been established, and the presence of IgM could simply indicate the reactivation of cytomegalovirus infection against the background of AOSD.

One reason for the delay in correct diagnosis was a false recovery during antibiotic therapy in a rural hospital. We believe this short remission was due to the patient taking high doses of paracetamol, up to 4,000 mg per day. The patient reported feeling much better with more than 1,500 mg of paracetamol per day, whereas a 75 mg injection of diclofenac in the hospital provided only weak and short-lived relief. A similar positive effect of paracetamol on AOSD has been described in the literature [16].

Currently, there are no evidence-based treatment regimens for AOSD with large studies; treatment is based on empirical observations and reviews by other researchers [17]. Our therapy choices were based on drug availability in our hospital. The dosage of intravenous prednisolone - 120 mg on the first day, 90 mg on the second day, and 60 mg on the third day - was based on our established treatment regimens for fevers of unclear origin. This therapy immediately reduced the fever and improved the patient's condition on the first day. Subsequently, the patient took methylprednisolone and methotrexate as baseline therapy. After discharge, she continued taking prednisolone 20 mg and methotrexate 10 mg for a month without side effects. Although the patient’s self-discontinuation of medication could have contributed to a recurrence of the disease, we unexpectedly found evidence of almost complete remission (except for slight increases in CRP, erythrocyte sedimentation rate, and alkaline phosphatase).

## Conclusions

This clinical case demonstrates that positive ANA and ANCA results can occur in AOSD. These antibodies can misleadingly suggest other autoimmune diseases, highlighting the importance of thorough data collection, including detailed patient history, for prompt and accurate diagnosis and treatment initiation.

## References

[1] Efthimiou P, Kontzias A, Hur P, Rodha K, Ramakrishna GS, Nakasato P (2021). Adult-onset Still's disease in focus: clinical manifestations, diagnosis, treatment, and unmet needs in the era of targeted therapies. Semin Arthritis Rheum.

[2] Feist E, Mitrovic S, Fautrel B (2018). Mechanisms, biomarkers and targets for adult-onset Still's disease. Nat Rev Rheumatol.

[3] Jiang L, Wang Z, Dai X, Jin X (2011). Evaluation of clinical measures and different criteria for diagnosis of adult-onset Still's disease in a Chinese population. J Rheumatol.

[4] Yamaguchi M, Ohta A, Tsunematsu T (1992). Preliminary criteria for classification of adult Still's disease. J Rheumatol.

[5] Pisetsky DS (2017). Antinuclear antibody testing - misunderstood or misbegotten?. Nat Rev Rheumatol.

[6] Merindol J, Levraut M, Seitz-Polski B, Morand L, Martis N (2023). Diagnostic significance of antineutrophil cytoplasmic antibody (ANCA) titres: a retrospective case-control study. RMD Open.

[7] Awad J, Farah R, Horn I (2007). Adult Still's disease despite the presence of positive antinuclear antibodies. Eur J Intern Med.

[8] Niranvichaiya S, Triwongwaranat D (2017). Diagnostic challenge: a report of two adult-onset Still's disease cases. Case Rep Dermatol Med.

[9] Lakshman H, Athwal PS, Gondi A, Dhillon S, Towfiq BA (2020). A case of adult-onset Still's disease with positive antinuclear antibodies. Cureus.

[10] Nagpure K, Raju P, Dube AH, Verma I, Kumbhalkar S (2024). Chronic adult-onset Still's disease with positive antinuclear antibodies: navigating diagnostic dilemmas and clinical implications. Cureus.

[11] Saghafi M, Sahebari M (2013). Does searching for antineutrophil cytoplasmic antibodies help with the diagnosis of Adult-onset Still's disease?. Rheumatol Int.

[12] Cozzi A, Papagrigoraki A, Biasi D, Colato C, Girolomoni G (2016). Cutaneous manifestations of adult-onset Still's disease: a case report and review of literature. Clin Rheumatol.

[13] Parks T, Smeesters PR, Curtis N, Steer AC (2015). ASO titer or not? When to use streptococcal serology: a guide for clinicians. Eur J Clin Microbiol Infect Dis.

[14] Aldehaim AY, Alnegheimish NA, Fathaddin AA (2020). Refractory urticaria with raised antistreptolysin O (ASO) titer: an intriguing case of adult-onset Still's disease. Am J Case Rep.

[15] Jia J, Shi H, Liu M (2019). Cytomegalovirus infection may trigger adult-onset Still's disease onset or relapses. Front Immunol.

[16] Kedzia A, Boldys A, Krysiak R, Szkrobka W, Okopien B (2009). Potential benefit of paracetamol administration in adult-onset Still's disease. Pol Arch Med Wewn.

[17] Giacomelli R, Caporali R, Ciccia F (2023). Expert consensus on the treatment of patients with adult-onset still's disease with the goal of achieving an early and long-term remission. Autoimmun Rev.

